# Cell adaptation of the extremophilic red microalga *Galdieria sulphuraria* to the availability of carbon sources

**DOI:** 10.3389/fpls.2022.978246

**Published:** 2022-09-15

**Authors:** Pablo Perez Saura, Malika Chabi, Amélie Corato, Pierre Cardol, Claire Remacle

**Affiliations:** Laboratory of Genetics and Physiology of Microalgae, InBios/Phytosystems Research Unit, University of Liège, Liège, Belgium

**Keywords:** *Galdieria sulphuraria*, heterotrophy, phototrophy, pigments, transcriptome, biomass composition, glucose, glycerol

## Abstract

Global energy demand and fossil fuels impact on climate can be partially managed by an increase in the use of biofuels for transports and industries. Biodiesel production is generally preceded by a transesterification process of the green biomass triacylglycerols that generates large amounts of glycerol as a by-product. In this study, the extremophilic red microalga *Galdieria sulphuraria* 074W was cultivated in heterotrophy. The microalgal growth parameters and biomass composition were compared when grown on an equivalent molar concentration of carbon of either glucose or glycerol as unique carbon source. The maximal biomass reached in these two conditions was not significantly different (∼2.5 g.L^–1^). Fatty acid profile, protein and storage carbohydrate contents were also statistically similar, irrespectively of the metabolized carbon source. We also observed that the pigment content of *G. sulphuraria* cells decreased during heterotrophic growth compared to photoautotrophic cultivated cells, and that this diminution was more important in the presence of glucose than glycerol: cells were yellowish in the presence of glucose and green in the presence of glycerol. The pigmentation was restored when glucose was totally consumed in the medium, suggesting that the presence of glucose repressed pigment synthesis. Based on this observation, a transcriptome analysis was performed in order to better understand the mechanisms involved in the loss of color mediated by darkness and by glucose in *G. sulphuraria*. Three conditions were analyzed: heterotrophy with glycerol or glucose and phototrophy. This allowed us to understand the transcriptional response of cells to light and dark environments both at the nuclear and chloroplast levels, and to show that transcription of gene families, acquired by horizontal gene transfer, such as sugar, amino acid, or acetate transporters, were involved in the response to the availability of different (in)organic sources.

## Introduction

Global energetic demand is rising continuously with more than 13,147 million tons of oil equivalent (Mtoe) in 2015 and more than 17,000 Mtoe forecast for 2040 (Dong et al., 2020). In 2020, primary energy consumption was still ensured at 80% by fossil fuels (British Petroleum, 2021; International Energy Agency, 2021). Apart from the fact that fossil fuels are not perennial resources, they have a high impact on global warming. One of the strategies to reduce this impact is the use of renewable energies, such as renewable biofuels (Field et al., 2020; International Energy Agency, 2021; Nazari et al., 2021). To reduce CO_2_ release to the atmosphere and face the global increase of temperature, IEA recommends that by 2050, around 25–65% of renewable fuels should be produced (International Energy Agency, 2021), with a majority of biodiesel, also called Fatty Acid Methyl Esters (FAMEs), intended in majority for the transport sector (Concawe., 2021; International Energy Agency, 2021).

Fatty Acid Methyl Esters are principally produced *via* a transesterification process performed on triglycerides found in animal fats, vegetable oils, waste cooking oils, or microalgae, that generates crude glycerol as a very cheap by-product (Sangaletti et al., 2013; Maneerung et al., 2016; Vivek et al., 2017). When 10 kg of FAMEs for biodiesel use are produced, about 1 kg of crude glycerol is generated and its purification into chemically pure glycerol is expensive and challenging (Sun and Chen, 2008; Ardi et al., 2015). Even if pure glycerol is a valuable material for many commercial applications, crude glycerol is, however, considered as a waste. Indeed, crude glycerol obtained from FAMEs production contains impurities (methanol and salts) that have an impact on metabolite production by microorganisms (Samul et al., 2014) or chemical use for industries (Kaur et al., 2020). However, different studies support the interest and the possibility to produce microorganism-valuable biocompounds with the use of crude glycerol as a substrate (Samul et al., 2014; Choi and Yu, 2015; Kaur et al., 2020). The red microalga *Galdieria sulphuraria* could be an interesting candidate for crude glycerol valorization because it has been reported to grow on glycerol (Gross and Schnarrenberger, 1995; Salbitani et al., 2021) unlike many other algae. In addition to glycerol, *G. sulphuraria* is able to grow on 26 different organic substrates (Gross and Schnarrenberger, 1995), many of which are usually not metabolized by heterotrophic microalgae. In contrast, glucose, the most widespread sugar found in nature can be metabolized by a large majority of microalgae (Perez-Garcia et al., 2011) and in the presence of glucose, *G. sulphuraria* can reach very high cell densities in heterotrophy (up to 100 g.L^–1^ in fed-batch and continuous flow cultures) (Schmidt et al., 2005; Graverholt and Eriksen, 2007). However, glucose and other sugars derived from food material have ethical limitations and are in general too expensive for large-scale biocompounds production (Li et al., 2007). An intriguing feature of *Galdieria* in heterotrophy is its ability to synthesize variable amounts of pigments, depending on the carbon source (Gross and Schnarrenberger, 1995).

*Galdieria sulphuraria* presents other interesting characteristics, such as the ability to grow up to 56°C (with an optimum at 42°C) and at very low pH (with an optimum at 2) (Hirooka and Miyagishima, 2016) which is explained by the conditions of its natural environment consisting of hot and acidic sulfurous springs of volcanic regions (Toplin et al., 2008; Ciniglia et al., 2014). These harsh growing conditions limit the presence of contaminating microorganisms, and even allow cultivation under non-sterile conditions (Pleissner et al., 2021; Scherhag and Ackermann, 2021). These characteristics are even more appropriate when the culture medium contains organic substrates that can be easily metabolized by contaminants such as fungi or bacteria that grow faster than microalgae. Moreover, *G. sulphuraria* has potential for biotechnological applications including bioremediation and biofuel production (Cheng et al., 2019; Cui et al., 2020; Somers et al., 2021), the capacity to resist to high salt concentrations, to recover heavy metals and nutrients from wastewaters (Ju et al., 2016; Delanka-Pedige et al., 2019; Scherhag and Ackermann, 2021; Sun et al., 2021), and to produce high added-value biocompounds such as phycocyanin and α-tocopherol (Carfagna et al., 2015; Bottone et al., 2019) and for nutritional applications (Graziani et al., 2013).

*Galdieria sulphuraria* is frequently studied for its capacity to grow heterotrophically (Cizkova et al., 2019). Contrary to the commonly used light-dependent strategy for microalgal growth, heterotrophy does not depend on climatic conditions, which is interesting in countries located in latitudes suffering weakness and scarcity of sunshine mostly during winter season. In addition, heterotrophic growth of microalgae can be conducted in fermenters that can be implemented in any type of surface and show higher cell-density yields than phototrophic cultivation systems (Perez-Garcia et al., 2011; Jareonsin and Pumas, 2021).

The nuclear genome sequence of *G. sulphuraria* was published in 2013. It is a small genome (13.7 Mb), characterized by gene families acquired by horizontal gene transfer (HGT) (Schönknecht et al., 2013). The transcriptional activity of some of these HGT gene families has been demonstrated in the acclimation process to temperature decrease (Rossoni et al., 2019).

Considering the large amounts of glycerol that are expected to be produced from FAMEs in the future, we aimed at evaluating how *G. sulphuraria* metabolizes it compared to glucose in heterotrophy. We showed that growth parameters were similar except the pigments content: cells were green with glycerol while they were yellowish with glucose. This led us to explore the transcriptome of cells grown in these two conditions and compare it to light conditions to get insight into the regulation of the pigment synthesis. This allowed us to understand the transcriptional response of cells to light and dark environments both at the nuclear and chloroplast levels, and to show that transcription of HGT genes is involved in the response to the availability of different (in)organic sources.

## Materials and methods

### Microalgal strain, culture media, and precultures

*Galdieria sulphuraria* strain 074 (hereafter 074W) was obtained from the algal Collection at the University Federico II (ACUF).^1^ Based on Pinto et al. (2003) and confirmed by the results presented here, this strain corresponds to the 074W isolate described in Gross and Schnarrenberger (1995), being white in the presence of glucose in heterotrophy. Prior to experimental cultivation, cells were maintained at 32°C on sterile 1% agar plates containing 2xGS modified Allen medium (Allen, 1959). One liter of medium contains: 300 mg (NH_4_)_2_SO_4_, 300 mg MgSO_4_⋅7H_2_O, 300 mg KH_2_PO_4_, 20 mg CaCl_2_⋅2H_2_O, 19.8 mg NaCl, 13.2 mg Fe-Na-EDTA, 5.72 mg H_3_BO_3_, 3.64 mg MnCl_2_⋅4H_2_O, 0.44 μg ZnSO_4_⋅7H_2_O, 2.1 mg (NH_4_)_6_Mo_7_O_24_⋅4H_2_O, CuSO_4_⋅5H_2_O, 0.05 mg NaVO_3_⋅4H_2_O, and 44 μg CoCl_2_⋅6H_2_O. pH was adjusted to 2.0 with 4N H_2_SO_4_ and the medium was sterilized by autoclaving for 20 min at 121°C. Liquid precultures were maintained in 250 ml flasks at 42°C in an incubator under continuous light (100 μmol_photon_.m^–2^.s^–1^) under constant shaking. Cells were then adapted to heterotrophy by transferring the flasks into a light-free incubator set at 42°C containing fresh 2xGS modified Allen medium supplemented with 5 g.L^–1^ of glucose or glycerol for 10 days. Adaptation step was performed two times before starting experimental cultures.

### Growth conditions and harvesting

Phototrophic cultures were seeded from phototrophic precultures in 500 ml flasks containing 140 ml of fresh 2xGS modified Allen medium. They were maintained at 42°C under constant shaking and illumination (100 μmol_photon_.m^–2^.s^–1^). Harvesting for RNA purification and pigment analysis in this condition were performed during exponential phase after 10 days of cultures at OD_800_ = 2.5 (OD, Optical Density). OD was determined measuring absorbance of the culture at a wavelength of 800 nm, using a UV-visible spectrophotometer (Perkin-Elmer lambda™ 265 UV/VIS, United States) in cuvettes of an optical path of 1 cm. Dilutions have been performed to reach an OD_800_ between 0.1 and 0.3. All phototrophic and heterotrophic culture experiments were performed in three independent biological replicates.

Heterotrophic cultures were seeded from two-times adapted heterotrophic precultures at a starting OD_800_ = 0.2. They were maintained under constant shaking in 500 ml flasks containing 140 ml of fresh 2xGS modified Allen medium supplemented with 25 mM of glucose or 50 mM of glycerol for 8 days in the dark in an incubator set at 42°C. Every day, samples were harvested for algal growth measurements and substrate quantification. After 2, 4, and 8 days of growth, samples were harvested for lipids, protein, and glycogen quantification. For pigment and RNA analysis, cells were collected after 2 days of growth from cells grown in three independent biological replicates.

### Algal growth measurements

Algal growth was determined daily by OD_800_, at which pigment absorbance is negligible, using a UV-visible spectrophotometer (Perkin-Elmer lambda™ 265 UV/VIS, United States) in cuvettes of an optical path of 1 cm. Dilutions have been performed to reach an OD_800_ between 0.1 and 0.3. OD measurements were extrapolated as dry biomass using an OD/biomass correlation. Biomass was estimated by centrifuging 25–50 ml of culture (3000×*g*; 3 min) once during exponential phase and once in stationary phase. Samples were washed twice in distilled water, centrifuged again, transferred in an aluminum cup pre-weighted and weighted after 48 h in a 70°C oven.

Specific growth rate (μ) expressed in day^–1^, was calculated as the slope of the linear regression of the natural log dry weight number as a function of time in exponential phase. Doubling time (Td), expressed in day was calculated as the natural log of 2 divided by the specific growth rate. Maximum biomass productivity expressed in g DW.L^–1^.day^–1^, was calculated by subtracting the dry biomass concentration after 3 days from dry biomass concentration after 4 days of growth. Biomass to substrate yield expressed in g DW.g substrate^–1^ was calculated dividing the dry biomass concentration after carbon source depletion to the initial carbon source concentration.

### Substrate quantification (carbon source, ammonium, phosphate)

Culture supernatants were harvested by centrifugation (16,000×*g*; 3 min) and pH was neutralized with small volumes of 10 M NaOH. Samples were 0.22 μm filtered and stored at −20°C before quantification.

Glucose and glycerol concentrations in the cultures were quantified by High Performance Liquid Chromatography (HPLC, Shimadzu, Tokyo, Japan) using an ion exclusion column Supelcogel C610-H (6% Crosslinked, 9 μm particle size, L × I.D. 30 cm × 7.8 mm, Sigma-Aldrich, Saint Louis, MI, United States) and a refractor index detector (RID-20A, Shimadzu). 40 μl of sample was charged in the column and elution was performed at 35°C with a flow of 0.5 ml.min^–1^ of H_3_PO_4_ 0.1% in isocratic mode as eluent during 35 min. Concentrations were determined based on the peak area of the chromatogram, compared to standard curves of known glucose or glycerol concentrations.

Ammonium concentration was determined using Berthelot reagent (Searle, 1984). In a 96-well plate, 20 μl of reagent (sodium nitroprusside 0.04%; phenol 3.5%; sodium hypochlorite 0.4%; NaOH 2%; trisodium citrate 38%) were added to 100 μl of sample. After 6 h of incubation, newly formed indophenol complexes absorbance was measured spectrophotometrically at 660 nm (Synergy Mx, Biotek Instruments, Inc., Winooski, VT, United States). Concentrations were calculated based on a standard curve of known NH_4_^+^ concentrations between 0 and 0.4 mM. The formula for ammonium concentration determination in the culture based on known concentrations was [NH]4+=A⁢660- 0.0804.112 (*r*^2^ = 0.9975). Samples were diluted in distilled water to an estimated NH_4_^+^ concentration between 0.05 and 0.4 mM if needed.

Phosphate concentration was measured spectrophotometrically based on a colorimetric method from Murphy and Riley (1962). In a 96-well plate, 50 μl of reagent (ascorbic acid 10%; ammonium heptamomybdate 2.5%; sulfuric acid 6 N; distilled water in proportions 1:1:1:2) were added to 50 μl of sample. After 30 min of incubation, absorbance was measured at 750 nm (Synergy Mx, Biotek Instruments, Inc., Winooski, VT, United States). Concentrations were calculated based on a standard curve of known PO_4_^–3^ concentrations between 0 and 0.75 mM. The formula for phosphate concentration determination in the culture based on known concentrations was $\left[P\,O_{4}^{-3}\right]=\frac{A\,750-0.050}{1.693}$(*r*^2^= 0.9985). Samples were diluted in distilled water to an estimated PO_4_^–3^ concentration between 0.15 and 0.75 mM if necessary.

### Pigments determination

#### Chlorophyll *a*, zeaxanthin, and β-carotene

Pigments were quantified using a reverse-phase HPLC method (Shimadzu, Tokyo, Japan) with a photodiode array detector SPD-M20A (PDA, Shimadzu, Tokyo, Japan). 2 ml of culture were centrifuged (16,000×*g*; 3 min) and the pellet was resuspended in dichloromethane/methanol (1:3, v/v). Glass beads were added to the sample and cells were disrupted through horizontal agitation in a TissueLyser II (Qiagen, Hilden, Germany) for 2 × 5 min at 30 Hz, with 5 min break on ice between the two lysing steps. After centrifugation (16,000×*g*; 3 min) and 0.22 μm filtration, 40 μl of samples were injected on a Nova-Pak C18 column (Nova-Pak silica column, 3.9 × 150 mm, 4 μm particle size, Waters, Milford, MA, United States). Elution was performed during 25 min at 25°C with a flow of 1 ml.min^–1^ using a gradient mode with three solvents: methanol 80% and ammonium acetate 100 mM (A), acetonitrile 90% (B), and ethyl acetate 100% (C). Gradient was: 0 min − 100% A; 0.5 min − 100% B; 1.1 min − 90% B + 10% C; 6.1 min − 65% B + 35% C; 11.5 min − 40% B + 60% C; 15.0 min − 100% C; 17.0 min − 100% A; 23.0 min − 100% A. Quantification was based on the peak area at 430 nm and compared to calibration curves obtained with pure pigments (DHI Lab Products, Denmark). Pigment content was then normalized on the dry biomass concentration.

#### Phycocyanin

Between 25 and 50 ml of culture were harvested, centrifuged (16,000×*g*; 3 min) and washed twice in 100 mM sodium phosphate buffer, pH 7.2, and resuspended in the same buffer. Glass beads were added to the sample and cells were disrupted through horizontal agitation in a TissueLyser II (Qiagen, Hilden, Germany) for 2 × 10 min at 30 Hz, with 5 min break on ice between the two lysing steps. Disrupted cells were centrifuged at 24,000×*g* for 90 min and the phycocyanin concentration was estimated spectrophotometrically (Perkin-Elmer lambda™ 265 UV/VIS, United States) as described in Kursar et al. (1983). Phycocyanin content was than normalized on soluble protein concentration (Bradford, 1976).

### Protein quantification

Total protein content was measured using the Bradford method (Bradford, 1976). 2 ml of culture were centrifuged (16,000×*g*; 3 min) and the pellet was resuspended in a detergent to solubilize all proteins (1% Triton X100; NaOH 0.1 M). Glass beads were added to the sample and cells were disrupted through horizontal agitation in a TissueLyser II (Qiagen, Hilden, Germany) for 2 × 10 min at 30 Hz with 5 min break on ice between the two lysing steps. In a 96-well plate, 10 μl of samples were added to 200 μl of Bradford reagent 1X. Absorbance of the samples was read at 595 nm (Synergy Mx, Biotek Instruments, Inc., Winooski, VT, United States). Concentrations were calculated based on a standard curve of known BSA concentrations diluted in the same solvent as the samples, between 0 and 0.5 mg.ml^–1^. Samples were diluted in the solubilization detergent (1% Triton X100; NaOH 0.1M) to an estimated protein concentration between 0.1 and 0.5 mg.ml^–1^ if necessary. Protein content was then normalized on the dry biomass concentration.

### Fatty Acid Methyl Esters quantification

2 to 4 ml of culture were centrifuged (16,000×*g*; 3 min) and the pellet was resuspended in chloroform/methanol (2:1, v/v). Glass beads were added to the sample and cells were disrupted through horizontal agitation in a TissueLyser II (Qiagen, Hilden, Germany) for 2 × 5 min at 30 Hz. Fatty Acid Methyl Esters (FAMEs) were generated from the microalgal biomass as described in Corato et al. (2022). FAMEs quantification was performed using gas chromatography (GC, Shimadzu, Tokyo, Japan) and a flame ionization detector (FID, Shimadzu) as mentioned in Gérin et al. (2020). 1 μl of sample was injected on a SGE BPX70 column (30 m × 0.25 mm × 0.25 μm) in “split” mode with a ratio of 10. Elution was ensured with a temperature gradient from 120 to 240°C at a speed of 4°C.min^–1^ with helium as carrier gas. The quantification of FAMEs was based on the peak area, using an external calibration curve realized with a FAMEs mix, suitable for microalgae fatty acids determination (Supelco37, Sigma-Aldrich, Saint Louis, MI, United States). Total fatty acid (FA) content was calculated by summing all the separated FAMEs concentrations. Fatty acid content was then normalized on the dry biomass concentration.

### Glycogen quantification

Polysaccharide accumulation was quantified. The polysaccharide of *G. sulphuraria* is a soluble “glycogen-type” polysaccharide, hereafter called glycogen (Shimonaga et al., 2008). Total glycogen content was determined with an enzymatic method using amyloglucosidase from *Aspergillus niger* (Megazyme, Ireland) to break the α-1,4-glycosidic bonds and a mix of hexokinase and glucose-6-phosphate dehydrogenase in the presence of ATP/NADP^+^ to generate one molecule of 6-phospho-gluconolactone and one molecule of NADPH per molecule of glucose. NADPH formation was measured at 340 nm spectrophotometrically (Synergy Mx, Biotek Instruments, Inc., Winooski, VT, United States). 2–4 ml of culture were centrifuged (16,000×*g*; 3 min) and the pellet was resuspended in 50 mM Tris-acetate buffer, pH 7.5. Glass beads were added to the sample and cells were disrupted through horizontal agitation in a TissueLyser II (Qiagen, Hilden, Germany) for 2 × 10 min at 30 Hz. Samples were then treated as described in Colpaert et al. (2021). Glycogen concentrations were calculated based on a standard curve of known glucose concentrations ranged between 0 and 1 g.L^–1^. Samples were diluted in distilled water to an estimated glucose concentration between 0.1 and 1 g.L^–1^ if necessary. Total glycogen content was calculated by summing all the separated FAMEs concentrations. Glycogen content was then normalized on the dry biomass concentration.

### Oxygen consumption rate measurements

Oxygen consumption rate was measured on cells harvested in exponential phase of growth using a Clark-type oxygen electrode (Hansatech, King’s Lynn, United Kingdom). 40 ml of cells were harvested at OD_800_ = 2.5 and concentrated by centrifugation (3500×*g*; 2 min) and resuspended in 10 ml of fresh medium to reach a OD_800_ = 10. After 1 h adaptation at 37°C, they were transferred into the oxygen electrode chamber set at 37°C (maximum controlled temperature of the device). Oxygen consumption rate was measured after 2–5 min in the dark as the slope of the linear regression of the oxygen consumption as a function of time. Values were normalized on the dry weight.

### Transcriptomic analysis

#### Sample collection and preparation

50 ml of culture of exponentially growing cells were centrifuged (3500×*g*; 2 min) and resuspended in a small volume of SDS-EB (2% SDS, 400 mM NaCl, 40 mM EDTA, 100 mM Tris–HCl, pH 8.0) for cell disruption through horizontal agitation in a TissueLyser II (Qiagen, Hilden, Germany) for 2 × 5 min at 30 Hz, in the presence of glass beads.

RNA extraction was performed as previously described (Remacle et al., 2010). Sample were sent to Novogene (United Kingdom) Company Limited for RNA sequencing and bioinformatic analysis.

#### RNA quantification and quality assessment

RNA integrity was assessed using the RNA Nano 6000 Assay Kit of the Bioanalyzer 2100 system (Agilent Technologies, Santa Clara, CA, United States).

#### Library preparation for transcriptome sequencing (Novogene Experimental Department)

Total RNA was used as input material for the RNA sample preparations. Briefly, mRNA was purified from total RNA using poly-T oligo-attached magnetic beads. Fragmentation was carried out using divalent cations under elevated temperature in First Strand Synthesis Reaction Buffer (5X). First strand cDNA was synthesized using random hexamer primer and M-MuLV Reverse Transcriptase (RNase H-). Second strand cDNA synthesis was subsequently performed using DNA Polymerase I and RNase H. Remaining overhangs were converted into blunt ends *via* exonuclease/polymerase activities. After adenylation of 3′ ends of DNA fragments, Adaptor with hairpin loop structure were ligated to prepare for hybridization. In order to select cDNA fragments of preferentially 370∼420 bp in length, the library fragments were purified with AMPure XP system (Beckman Coulter, Beverly, United States). Then PCR was performed with Phusion High-Fidelity DNA polymerase, Universal PCR primers and Index (X) Primer. At last, PCR products were purified (AMPure XP system) and library quality was assessed on the Agilent Bioanalyzer 2100 system.

#### Clustering and sequencing (Novogene Experimental Department)

The clustering of the index-coded samples was performed on a cBot Cluster Generation System using TruSeq PE Cluster Kit v3-cBot-HS (Illumina) according to the manufacturer’s instructions. After cluster generation, the library preparations were sequenced on an Illumina Novaseq platform and 150 bp paired-end.

#### Data analysis (Novogene Experimental Department)

##### Quality control

Raw data (raw reads) of fastq format were firstly processed through in-house perl scripts. In this step, clean data (clean reads) were obtained by removing reads containing adapter, reads containing ploy-N and low quality reads from raw data. At the same time, Q20, Q30 and GC content the clean data were calculated. All the downstream analyses were based on the clean data with high quality.

##### Reads mapping to the reference genome

Reference genome and gene model annotation files were downloaded from genome website directly. Index of the reference genome was built using Hisat2 v2.0.5 and paired-end clean reads were aligned to the reference genome using Hisat2 v2.0.5. We selected Hisat2 as the mapping tool for that Hisat2 can generate a database of splice junctions based on the gene model annotation file and thus a better mapping result than other non-splice mapping tools.

##### Quantification of gene expression level

FeatureCounts v1.5.0-p3 was used to count the reads numbers mapped to each gene. FPKM of each gene was calculated based on the length of the gene and reads count mapped to this gene. FPKM, expected number of Fragments Per Kilobase of transcript sequence per Millions base pairs sequenced, considers the effect of sequencing depth and gene length for the reads count at the same time, and is currently the most commonly used method for estimating gene expression levels.

##### Differential expression analysis

Differential expression analysis of two conditions/groups (three biological replicates per condition) was performed using the DESeq2R package (1.20.0). DESeq2 provide statistical routines for determining differential expression in digital gene expression data using a model based on the negative binomial distribution. The resulting *P*-values were adjusted using the Benjamini and Hochberg’s approach for controlling the false discovery rate. Genes with an adjusted *P*-value ≤ 0.05 found by DESeq2 were assigned as differentially expressed.

##### Gene Ontology and Kyoto Encyclopedia of Genes and Genomes enrichment analysis of differentially expressed genes

Gene Ontology (GO) enrichment analysis of differentially expressed genes was implemented by the clusterProfiler R package, in which gene length bias was corrected. GO terms with corrected *P*-value less than 0.05 were considered significantly enriched by differential expressed genes. Kyoto Encyclopedia of Genes and Genomes (KEGG) is a database resource for understanding high-level functions and utilities of the biological system, such as the cell, the organism and the ecosystem, from molecular-level information, especially large-scale molecular datasets generated by genome sequencing and other high-through put experimental technologies.^2^ We used clusterProfiler R package to test the statistical enrichment of differential expression genes in KEGG pathways.

## Results

### Growth parameters, nutrient consumption, and biomass composition

**FIGURE 1:**
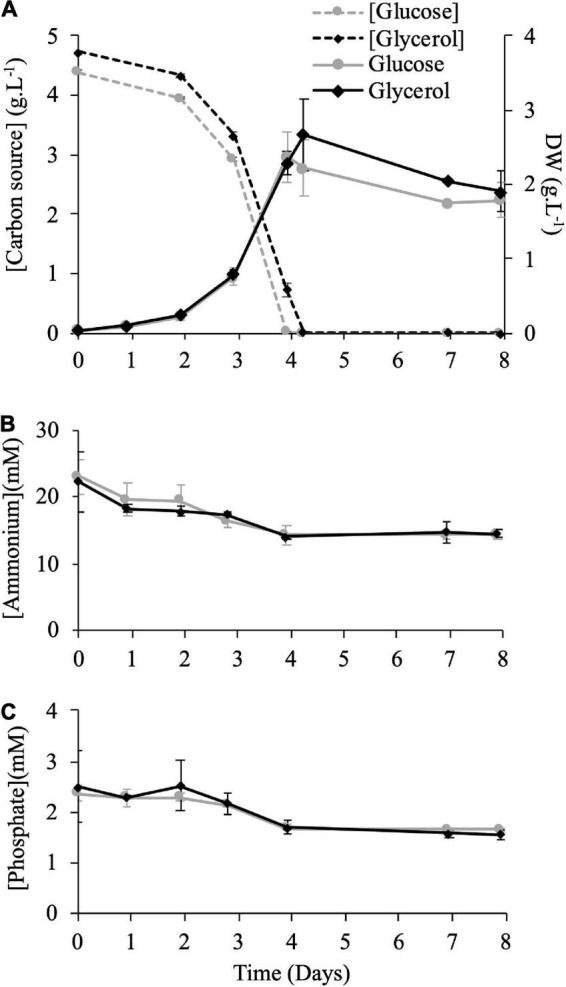
Biomass dry weight and nutrient concentrations in the medium of *Galdieria sulphuraria* cells grown in heterotrophy in the presence of glucose or glycerol as carbon source. **(A)** Dry Weight (DW) evolution (solid lines, secondary axis) and carbon source concentration in the medium (dashed lines, primary axis) over time (days). Glucose: gray circles; Glycerol: black diamonds. Data are expressed in g.L^–1^ of culture. Graphs **(B,C)** show ammonium and phosphate concentrations in the culture in mM over time (days). Data are presented as means of three independent biological replicates. Error bars represent standard deviation of the mean (±SD).

Our aim was to characterize the heterotrophic growth of *Galdieria* cells cultivated on glycerol and compare to growth on glucose, taken as a widely used carbon source reference. The cultures were maintained under heterotrophy at 42°C. 150 mM carbon atoms were present in both cases, corresponding to 25 mM glucose (4.5 g/L) and 50 mM glycerol (4.6 g/L). Cells were adapted to the carbon source during two consecutive cultivation periods before the analysis. As shown in Figure 1A, growth was similar in both cases, and the stationary phase was reached at day 4 upon depletion of the carbon source. The nitrogen (ammonium) and phosphate sources were not depleted at day 4 (Figures 1B,C), confirming that the carbon source is the main nutrient responsible for growth arrest.

**FIGURE 2:**
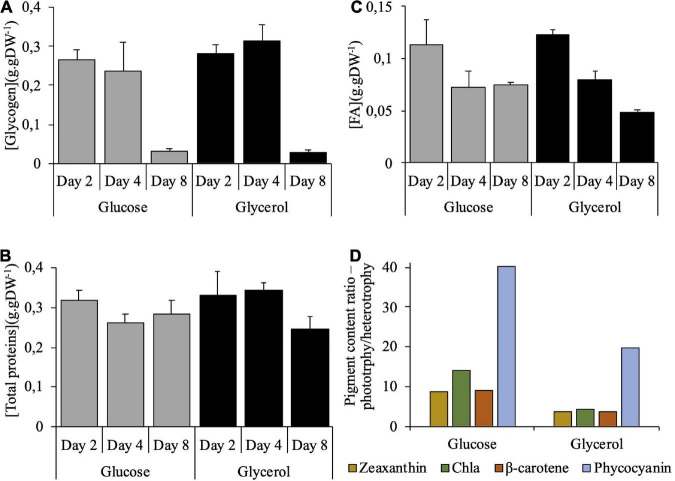
Biomass composition of *Galdieria sulphuraria* cells grown in heterotrophy in the presence of glucose or glycerol. Glucose (gray bars) or glycerol (black bars). Graphs **(A–C)** show the total glycogen, proteins, and fatty acids content expressed in g.g DW^–1^, respectively. Day 2: exponential phase; Day 4: early stationary phase; Day 8: late stationary phase (last day of culture). Data are presented as means of three independent biological replicates. Error bars represent standard deviation (±SD). Bar chart **(D)** shows the phototrophy to heterotrophy (glucose or glycerol as substrate) pigment content ratio in exponential phase (Day 2). Pigments shown for each data series are, from left to right, zeaxanthin, chlorophyll *a* (Chla), β-carotene, and phycocyanin. Data are presented as the mean from three independent biological replicates of *G. sulphuraria* grown in phototrophy divided by the mean from three independent biological replicates grown in heterotrophy.

The different growth parameters such as doubling time, maximum biomass, maximum productivity, were similar between glycerol and glucose (Table 1) and also in the range of those found in the literature (Schmidt et al., 2005; Sloth et al., 2006; Graverholt and Eriksen, 2007; Rahman et al., 2020; Scherhag and Ackermann, 2021). Whole cell respiration rates were also similar at mid-exponential phase (Table 1) and the yield of biomass to substrate was comparable for the two carbon sources (Table 1), suggesting that the efficiency of carbon assimilation was the same in both conditions. To check whether the carbon allocation inside the cells would be different according to the carbon source, the biomass composition was investigated at three timepoints: at day 2 (exponential phase), day 4 (start of the stationary phase), and day 8 (stationary phase) (Figure 2). The three components analyzed, glycogen, protein and fatty acids, presented the same profile of accumulation on both carbon sources. Glycogen content represented ∼30% of DW until day 4 (Figure 2A). It dropped at day 8, constituting only ∼10 % of the amount found at the beginning of the growth. Protein content also represented ∼30% of DW and remained high until day 8, still constituting between ∼70 and 80% of the initial content (Figure 2B). FAME analysis showed that fatty acid content represented ∼11–12% of DW at the beginning of the growth, which is higher compared to values of the literature (∼4%, López et al., 2019; Mozaffari et al., 2019). The content was reduced by one third at day 4, and remained stable until day 8 (Figure 2C). Fatty acid composition was analyzed. The major fatty acids accumulated were saturated fatty acids [mainly palmitic (C16:0) and stearic (C18:0) acids, Supplementary Table 1] which represented ∼55% of the total amount at day 2 and ∼45% at day 8. The monounsaturated fatty acids (mainly oleic acid, C18:1 ω-9, Supplementary Table 1) increased during growth, to reach ∼18% of the total fatty acid at day 8 while the polyunsaturated fatty acids (mainly linoleic and linolenic acid) constituted ∼35% of the total fraction during the entire course of the growth.

**TABLE 1:** Comparison of specific growth rate and biomass substrate yield of *Galdieria sulphuraria* heterotrophic growth on different substrates.

Strain	Culture condition	Carbon source	Concentration (g.L^–1^)	Specific growth rate (μ) (day^–1^)	Doubling time (Day)	Max biomass (g.L^–1^)	Max productivity (gDW.L^–1^. d^–1^)	Oxygen consumption rate (mmolO_2_. min^–1^.gDW^–1^)	Sugar to biomass conversion (gDW.gsugar^–1^)	References
074W	500 ml Shake flasks	Glucose	4.5	1.10 ± 0.03	0.63 ± 0.02	2.37 ± 0.34	1.62 ± 0.22	9.56 ± 0.28	0.56 ± 0.08	This study
074W	500 ml Shake flasks	Glycerol	4.6	1.08 ± 0.03	0.64 ± 0.02	2.67 ± 0.48	1.50 ± 0.10	9.78 ± 0.25	0.57 ± 0.05	This study
074G	500 ml Shake flasks	Glucose	4.5	1.10	0.63	/	/		0.48	Schmidt et al., 2005
074G	500 ml Shake flasks	Glucose + fructose	52.5	1.44	0.48	/	/		0.42	Schmidt et al., 2005
SAG 21.92	500 ml Shake flasks	Sucrose, glucose, and fructose	36	1.44	0.48	/	/		0.63	Scherhag and Ackermann, 2021
SAG 21.93	500 ml Shake flasks	Sucrose, glucose, and fructose	6	1.53	0.45	/	/		0.64	Scherhag and Ackermann, 2021
074G	3 L Bioreactor	Glucose	10	1.26	0.55	/	/		0.46	Graverholt and Eriksen, 2007
074G	500 ml Shake flasks	Glucose	5	1.20	0.58	2.5	/		0.52	Sloth et al., 2006
074G	500 ml Shake flasks	Glycerol	5	1.20	0.58	3.5	/		0.70	Sloth et al., 2006
074G	1 L Bioreactor	Glucose	10	0.72	0.96	4.7	/		0.47	Rahman et al., 2020

**FIGURE 3:**
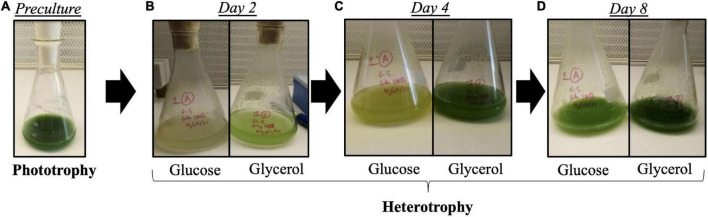
Images of *Galdieria sulphuraria* cultures. Cultures in phototrophy as a preculture **(A)**, or in heterotrophy after 2 days **(B)**, 4 days **(C)**, and 8 days **(D)** in the presence of 25 mM of glucose or 50 mM of glycerol as carbon source. Yellow color of the culture mediated by the pigment loss can clearly be observed during exponential phase **(B)** only when glucose is present. After glucose depletion at stationary phase **(D)**, a return of the green color is observed.

A major difference was observed at the level of pigment content: as shown in Figure 3, *Galdieria* cells cultivated under glucose were yellowish at day 2 and day 4 while those cultivated under glycerol were green. On day 8, the pigmentation was restored in glucose-grown cells, when glucose was totally consumed. The phototrophy to heterotrophy (glucose or glycerol as substrate) pigment (chlorophyll *a*, zeaxanthin, beta-carotene, and phycocyanin) content ratios were calculated (Figure 2D) from absolute pigment content values (Supplementary Figure 1). Pigment ratios are above 1 for all the pigments, meaning that more pigments are present in phototrophy than in heterotrophy as expected. Chlorophyll *a*, zeaxanthin and beta carotene content ratios show a 2-fold increase in phototrophy compared to glycerol condition in heterotrophy, whereas the ratios show between 8- and 14-fold increase in phototrophy compared to glucose condition in heterotrophy. The phycocyanin content shows a 20-fold increase in phototrophy compared to glycerol and a 40-fold increase when compared to glucose. It can thus be concluded that heterotrophy inhibits pigment synthesis, and that the inhibition is stronger in the presence of glucose than with glycerol.

### Transcriptomics analysis

In order to decipher the regulation underlying the inhibition of pigment synthesis in the dark, the transcriptome response of *Galdieria* cells grown in heterotrophy with glucose or glycerol was compared. In addition, the transcriptome response of cells grown in phototrophy was also added in the analyses as a control for full pigment synthesis and the lack of organic carbon source for growth.

#### Data summary and global gene expression profiling analysis

For that purpose, RNA was extracted in three biological replicates from cultures at mid-exponential phase in heterotrophy with glycerol or glucose and in phototrophy. RNA-seq data were obtained and the sequencing data are presented in Supplementary Table 2. The raw datasets generated for this study are deposited on NCBI [Sequence Read Archive (SRA), PRJNA854810^3^ ].

**FIGURE 4:**
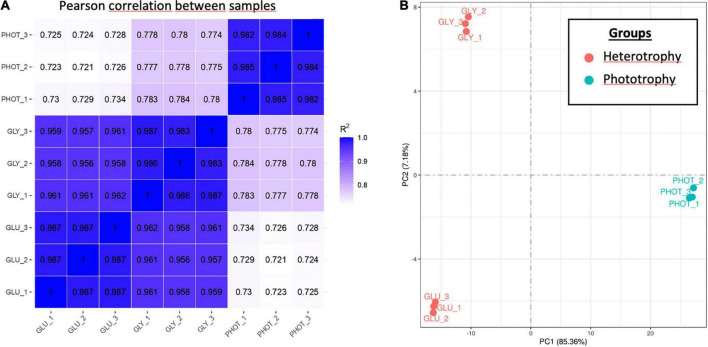
Pearson correlation (PC) and principal component analysis (PCA) of the RNA-seq dataset for nine independent *Galdieria sulphuraria* samples harvested in exponential phase. **(A)** PC and **(B)** PCA made on the gene expression values (FPKM) of all samples. Samples are shown as independent biological replicates of three different culture conditions. PHOT_1, PHOT_2, and PHOT_3 are samples of *G. sulphuraria* grown in phototrophy without any carbon source. GLU_1, GLU_2, and GLU_3 are samples of *G. sulphuraria* grown in heterotrophy in the presence of 25 mM of glucose. GLY_1, GLY_2, and GLY_3 and samples of *G. sulphuraria* grown in heterotrophy in the presence of 50 mM of glycerol. In panel **B**, blue color dots represent samples grown in phototrophy, while red color mark samples grown in heterotrophy.

Comparison of global gene expression profiles between each pair of samples revealed that the biological replicates were highly correlated with each other (*r* > 0.98), which suggested experimental reliability. In addition, the samples cultivated on glycerol were highly correlated with those on glucose (*r* ∼ 0.95), while the three samples cultivated under phototrophy exhibited lower correlation rates with the two other sets of samples (*r* ∼ 0.78) suggesting that the presence of light influences more the global expression than the source of carbon in the dark (Figure 4A).

The principal component analysis (PCA) on the gene expression values (FPKM) of all samples showed that the three biological replicates clustered together for each condition with PC1 (85% of the gene expression variance) explaining the dependence to the light and PC2 (7% of the gene expression variance) explaining the dependence to the carbon source under heterotrophy (Figure 4B).

#### Differential expression analysis and biological pathways unique to the response to the different growth conditions

##### Comparison between glucose and glycerol samples grown in heterotrophy

**FIGURE 5:**
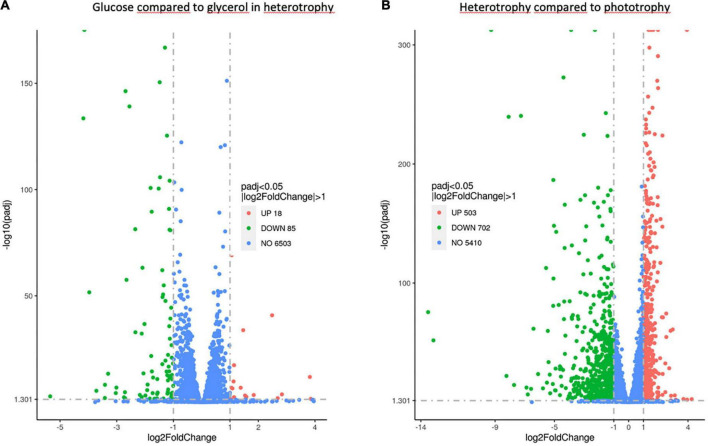
Vulcano plot of the RNA-seq results. Differential expression analysis of *Galdieria sulphuraria* genes when grown in heterotrophy in the presence of glucose compared to glycerol **(A)** and in heterotrophy (glucose and glycerol) compared to phototrophy **(B)** RNA-seq data sets. Red and green points mark the genes that are up or down regulated, respectively (*p*adj < 0.05 and |log2FoldChange| > 1) while blue points mark not differentially expressed genes (*n* = 3). The x-axis shows log2FoldChange in gene expression and the y-axis shows the –log10 ajust *p*.value. *P* threshold in reported as horizontal gray dashed line (=0.05).

Differentially expressed genes (DEGs) [adjusted *p* < 0.05 and log2(fold change) > 1] were identified in the samples grown in heterotrophy with glucose or glycerol (Figure 5A). The expression of 18 genes was found significantly upregulated in the samples cultivated with glucose compared to those cultivated with glycerol (Supplementary Table 3). Two hypothetical transcription factors were identified, one with a myb_DNA binding domain (Gasu_31830) and the other with a basic-leucine zipper domain (Gasu_38680) as well as a sugar transporter (Gasu_53180) in addition to many hypothetical genes with unknown function.

**FIGURE 6:**
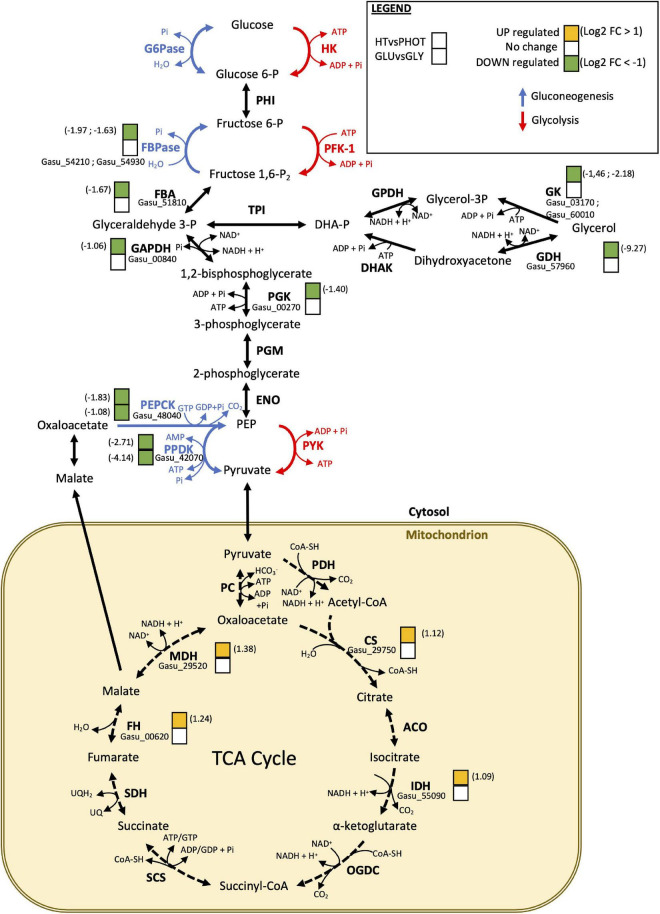
Schematic representation of glycolysis, gluconeogenesis, and tricarboxylic acid (TCA) cycles. Solid red and blue arrows mark specific reactions of glycolysis and gluconeogenesis, respectively. Cytosol is in white and mitochondrion is in light brown. Enzymes are highlighted and intermediated metabolites are shown in normal letters. Enzymes encoded by differentially expressed genes are marked with two overlapped squares. The one on the bottom refers to glucose compared to glycerol differentially expressed genes and the one on the top refers to heterotrophy compared to phototrophy differentially expressed genes. Green color is for downregulated genes, yellow color for overexpressed genes and white color means no significant differences in gene expression. Log2FoldChange is written on the right or on the left size of the squares when differences are observed. Transcript names are written above enzyme abbreviations. Abbreviations for enzymes: G6Pase, glucose-6-phosphatase; HK, hexokinase; FBPase, fructose 1,6-biphosphatase; PFK-1, phosphofructokinase-1; FBA, aldolase; TPI, triose-phosphate isomerase; GPDH, glycerol 3-phosphate dehydrogenase; GK, glycerol kinase; DHAK, dihydroxyacetone kinase; GDH, glycerol dehydrogenase; GAPDH, glyceraldehyde-phosphate dehydrogenase; PGK, phosphoglycerate kinase; PGM, phosphoglycerate mutase; ENO, enolase; PEPCK, phosphoenolpyruvate carboxykinase; PPDK, pyruvate phosphate dikinase; PYK, pyruvate kinase; PDH, pyruvate dehydrogenase; PC, pyruvate carboxylase; CS, citrate synthase; ACO, aconitase; IDH, isocitrate dehydrogenase; OGDC, α-ketoglutarate dehydrogenase; SCS, succinyl-CoA synthetase; SDH, succinate dehydrogenase; FH, fumarase; MDH, malate dehydrogenase.

**FIGURE 7:**
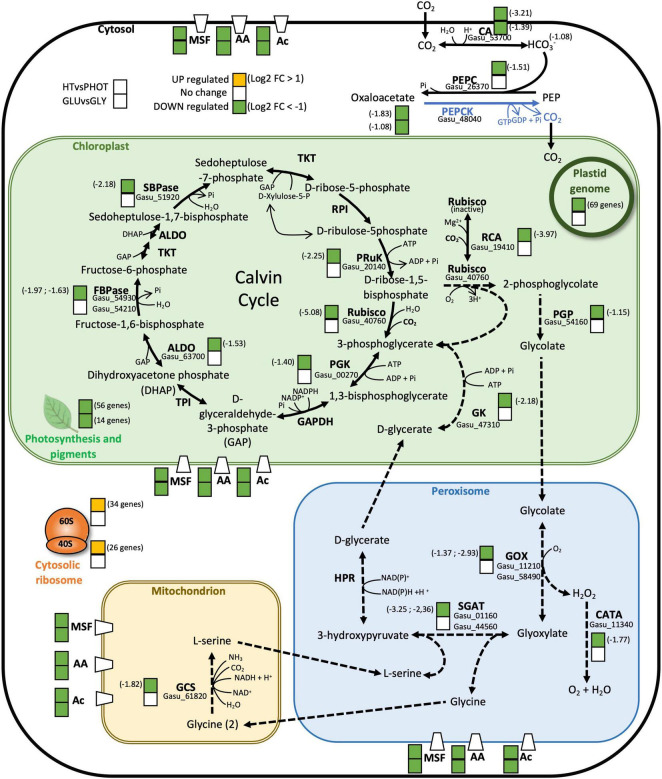
Schematic representation of the carbon concentrating (CCM) and photorespiration pathways (solid and dashed arrows, respectively). Cytosol is in white, chloroplast is in green, peroxisome is in blue, mitochondrion is in light brown, plastid genome in dark green, and ribosome is in orange. Leaf drawing shows the number of genes that are differentially expressed in photosynthesis related enzymes and pigment biosynthesis. Enzymes are highlighted and intermediated metabolites are shown in normal letters. Enzymes encoded by differentially expressed genes are marked with two overlapped squares. The one on the bottom refers to glucose compared to glycerol differentially expressed genes and the one on the top refers to heterotrophy compared to phototrophy differentially expressed genes. Green color is for downregulated genes, yellow color for overexpressed genes, and white color means no significant differences in gene expression. Log2FoldChange is written on the right or on the left size of the squares when differences are observed. Gene names are written above enzymes abbreviation. Blue arrow shows the limiting step of gluconeogenesis liberating carbon dioxide. Abbreviations for enzymes: ALDO, aldolase (fructose-bisphosphate aldolase); FBPase, fructose-1,6-bisphosphatase; GAPDH, glyceraldehyde-3-phosphate dehydrogenase; PGK, phospho-glycerate kinase; PRuK, phosphoribulokinase; RPE, ribulosephosphate 3-epimerase; RPI, ribose 5-phosphate isomerase; Rubisco, ribulose-1,5-bisphosphate carboxylase/oxygenase; SBPase, sedoheptulose-1,7-bisphosphatase; TKT, transketolase; TPI, triose-phosphate isomerase; PGP, phosphoglycolate phosphatase; CA, carbonic anhydrase; PEPC, phosphoenolpyruvate carboxylase; PEPCK, phosphoenolpyruvate carboxykinase; CATA, catalase; GOX, glycolate oxidase; SGAT, serine/glyoxylate transaminase; HPR, hydroxypyruvate reductase; GK, glycerate kinase; GCS, glycine cleavage system; RCA, rubisco activase. Transporter proteins are marked with white trapezes crossing the membranes and their abbreviations are: MSF, major facilitator superfamily; AA, amino acid transporters; Ac, acetate transporters.

Eighty-five genes were downregulated in the samples cultivated with glucose compared to those cultivated with glycerol (Supplementary Table 3). Transcripts of two genes encoding enzymes that catalyze the production of phosphoenolpyruvate from pyruvate were in decreased amounts in glucose compared to glycerol: Gasu_42070 encoding pyruvate phosphate dikinase (PPDK)/phosphoenolpyruvate synthase (−4.14) and Gasu_48040 (−1.07) encoding phosphoenolpyruvate carboxykinase (PEPCK), with Gasu_42070 being the most downregulated transcript of the list. These two enzymes belong to gluconeogenesis (Figure 6), which would suggest that this pathway is downregulated in the presence of glucose contrary to the situation with glycerol, although proteomic data are missing to confirm this conclusion. Indeed, glycerol is a preferential substrate for gluconeogenesis (Shah and Wondisford, 2020; Figure 6). In addition to glycerol, gluconeogenesis is also supplied by amino acids (Shah and Wondisford, 2020). Interestingly five genes encoding amino acid transporters and amino acid permeases were downregulated in glucose compared to glycerol (Figure 7 and Supplementary Table 4). Ten genes encoding transporters from the Major Facilitator Superfamily (MSF) with proposed sugar transporter activity were also downregulated in the presence of glucose while only one was upregulated (Figure 7 and Supplementary Table 4). Four genes encoding putative acetate transporters were similarly downregulated in glucose (Figure 7 and Supplementary Table 4). Lastly, 11 genes involved in photosynthesis were downregulated in glucose. As mentioned above, these variations of transcript abundance cannot be directly translated into regulation of metabolism in the absence of comparative proteomic and functional analyses. Although the pigment content varies between glucose and glycerol grown cells (Figure 3 and Supplementary Figure 1), expression of genes involved in chlorophylls, phycocyanin or carotenoid synthesis was not significantly modified between these two conditions (Supplementary Table 4).

Gene Ontology (GO) and KEGG pathway analyses on all the DEGs were analyzed and the significant processes (*p*adj < 0.05) are shown (Supplementary Figure 2 and Supplementary Tables 5, 6). They revealed 17 GO terms located in biological processes (BP, 5), cellular components (CC, 10), and molecular function (MF, 2). The KEGG enrichment identified two pathways related to photosynthesis (photosynthesis and photosynthesis antenna).

##### Comparison between samples grown in heterotrophy and phototrophy

DEGs [adjusted *p* < 0.05 and log2(fold change) > 1] were identified in the samples cultivated in heterotrophy versus phototrophy (Figure 5B). We thus searched for genes whose expression is similar in heterotrophy, whatever the sugar considered and significantly differs in phototrophy. The expression of 503 genes was upregulated in heterotrophy compared to phototrophy (Supplementary Table 3). As proteomic data were available for comparison between heterotrophy and phototrophy (Curien et al., 2021), we calculated the protein ratios between these two conditions in Supplementary Table 4. The transcript and protein ratios usually followed the same trend, which would suggest an impact on the affected metabolisms. Considering the energetic mitochondrial metabolism, the expression of most of the genes encoding subunits of the complexes of the respiratory chain was unmodified, except for two components of NADH:ubiquinone oxidoreductase (complex I), two components ATP synthase (complex V) and an assembly factor of this last complex (Supplementary Table 4). The expression of the alternative oxidase (Gasu_36260) was upregulated. This enzyme is known to be subject to transcriptional regulation in algae and land plants, upon various stresses (Baurain et al., 2003; Molen et al., 2006; Selinski et al., 2018) to avoid electrons accumulation in the respiratory chain and ROS production, which is in agreement with its higher expression in heterotrophy for *G. sulphuraria*. An increased expression of four genes (out of 8) encoding enzymes of the Tricarboxylic Acid Cycle (TCA) has been found, which would suggest a transcriptional upregulation of this cycle in heterotrophy (Supplementary Table 4 and Figure 6). Lastly, a significant number of genes encoding proteins of the large (35 for the 60S) and small (27 for the 40S) subunits was upregulated (Supplementary Table 4 and Figure 7).

The expression of 702 genes was downregulated in heterotrophy versus phototrophy (Supplementary Table 3). The most downregulated gene (−13.51) encodes a putative acetate transporter (Gasu_57950) and like in the comparison glucose/glycerol in heterotrophy, a large number of putative transporters were found downregulated in heterotrophy versus phototrophy: 29 amino acid transporters/permeases, 36 transporters of MSF, 7 acetate transporters (Figure 7 and Supplementary Table 4).

As expected, a large number of genes of photosynthesis were downregulated in heterotrophy and the downregulation also comprises genes involved in pigment synthesis (chlorophylls, phycocyanin, and carotene, Supplementary Table 4) and components of the Calvin cycle (Supplementary Table 4 and Figure 7). Considering the poor availability of CO_2_ especially at pH 2 and at 42°C (Gross, 1999), we also found downregulation of transcripts encoding the pseudo carbon-concentrating mechanism of red microalgae (Rademacher et al., 2017; Curien et al., 2021) and photorespiration in heterotrophy compared to phototrophy (Figure 7 and Supplementary Table 4). The Rubisco activase, known to be important in low CO_2_ condition in microalgae (Pollock et al., 2003) was also strongly downregulated in heterotrophy. The second most downregulated gene (−9.26) encodes a glycerol dehydrogenase/iron containing alcohol dehydrogenase (Gasu_57960), which belongs to a diverse and ancient protein family of iron dehydrogenases (FeADH), present in bacteria and microorganisms but absent in land plants (Gaona-López et al., 2016). The enzyme shows the GlyGlyGlyXXXAsp amino acid motif for cofactor [NAD(P)+] binding as well as several Asp and His amino acid residues as putative Fe-ligands. In addition to their role in alcohol assimilation, some FeADHs have a critical role in oxidative stress in bacteria (Lin et al., 2021), which could explain the downregulation of Gasu_57960 in heterotrophic condition compared to phototrophy where light oxidative stress is present (Supplementary Table 4). In addition, genes encoding components of gluconeogenesis were also downregulated in heterotrophy (Supplementary Table 4 and Figure 6).

Lastly, two nucleus-encoded RNA polymerase sigma factors (Gasu_28760, Gasu_35420) of the chloroplast-encoded RNA polymerase were downregulated in heterotrophy (−2.00, −1.94) and 69 genes of the chloroplast genome, including two components of the chloroplast-encoded RNA polymerase (Gasu_40590, Gasu_40330) (Figure 7 and Supplementary Table 4). This suggests that the chloroplast genome is subject to transcriptional regulation and that chloroplast transcripts of *G. sulphuraria* are polyadenylated and can be trapped by poly-T primers like the nucleus-encoded transcripts as it was demonstrated for the chloroplast-encoded transcripts of the green microalga *Chlamydomonas reinhardtii* (Shi et al., 2016).

Gene Ontology (GO) and KEGG pathway analyses on all the DEGs revealed 32 GO terms and 6 biosynthetic pathways (Supplementary Figure 2 and Supplementary Tables 5, 6). The GO terms are located in biological processes (BP, 13), cellular components (CC, 14) and molecular function (MF, 5) in which the BP of photosynthesis, translation and peptide biosynthesis are at the top. The top 3 KEGG pathways are related to ribosome, photosynthesis and photosynthesis-antenna proteins.

## Discussion

The first aim of this study was to understand the physiology of *Galdieria* cells grown in heterotrophy when glucose or glycerol are used as reduced carbon source. All the parameters analyzed were similar irrespective of the carbon source, except the pigment content. This content was significantly higher in glycerol than in glucose, although it remained significantly much lower than in phototrophy. This led us to perform a transcriptomics analysis in three conditions: glucose or glycerol as carbon source in heterotrophy and phototrophy. This analysis showed that 11 transcripts related to photosynthesis were significantly downregulated in glucose when glucose- and glycerol-grown cells were compared. The decrease was even more pronounced when comparing heterotrophy and phototrophy since in that case 36 genes were downregulated. The expression of genes involved in pigment synthesis was also downregulated in heterotrophy compared to phototrophy while the difference was not significant when the comparison was made between cells grown in glucose and glycerol in heterotrophy (Supplementary Table 4). The difference between glycerol- and glucose-grown cells could only be detected at the level of pigment synthesis when each condition was separately compared to phototrophy (Glu/PHOT and Gly/PHOT, Supplementary Table 4). A significant downregulation of genes involved in pigment synthesis (chlorophylls, phycocyanin, and carotenoids) and in photosynthesis could be seen in both cases (Glu/PHOT and Gly/PHOT), with generally a stronger level of downregulation for cells grown in glucose than for cells grown in glycerol (Supplementary Table 4). It could thus be concluded that heterotrophy and glucose in particular exerts a negative control on the expression of genes involved in photosynthesis, and pigment synthesis compared to phototrophy. The role of glucose had been already studied in the light (mixotrophy). This sugar exerts a negative control on photosynthesis activity in *Galdieria* (Oesterhelt et al., 2007; Curien et al., 2021) and other microalgae (Martínez and Orús, 1991) in the absence of supplemental CO_2_. In the green microalga *Chromochloris zofingiensis*, this sugar regulates photosynthesis and pigment synthesis at transcriptional level in mixotrophy, with hexokinase playing a critical role for this regulation (Roth et al., 2019). In the dark, glucose addition is responsible for the decrease/loss of pigmentation in *G. sulphuraria* (Gross and Schnarrenberger, 1995; Schönknecht et al., 2013). Heterotrophy whatever the carbon source considered is also known to reduce pigment content in microalgae including *G. sulphuraria* (Martínez and Orús, 1991; Mozaffari et al., 2019). In *G. sulphuraria* and other microalgae, this decrease could be or not accompanied by alteration of the chloroplast ultrastructure (Pellegrini, 1980; Tischendorf et al., 2007; Mozaffari et al., 2019).

The results presented here also show that heterotrophy negatively regulates the expression of the chloroplast-encoded genes. Amongst the seven nucleus-encoded chloroplast RNA polymerase sigma factors (Gasu_29800, Gasu_17390, Gasu_43280, Gasu_54330, Gasu_44980, Gasu_28760, Gasu_35420) found in the DEGs between heterotrophy and phototrophy, two (Gasu_28760, Gasu_35420) were significantly downregulated, which would suggest that they are required for controlling expression of the chloroplast genome in heterotrophy. In *Cyanidoschyzon merolae*, a red microalga very close to *G. sulphuraria*, a MYB2 transcription factor represses the expression of the nuclear-encoded chloroplast RNA polymerase sigma factor gene SIG2, which results in the downregulation of the expression of the chloroplast-encoded phycobilisome genes (Kawase et al., 2017). Only one myb-family related factor was found upregulated in heterotrophy (Gasu_46180, +1.12), which could thus be considered as a good candidate for the repression of sigma factors controlling chloroplast-encoded RNA polymerase.

A similar trend was very often observed between the transcript ratios determined in this study and the protein ratios calculated from proteomic data (Curien et al., 2021) when heterotrophy and phototrophy were compared (Supplementary Table 4). This observation is valid for chloroplast- and nucleus-encoded genes/proteins, meaning that when a transcript is downregulated in heterotrophy compared to phototrophy, the relative abundance of the corresponding protein is reduced in that condition too. This would suggest that post-transcriptional regulation is not frequent. Concerning transcripts of chloroplast-encoded genes, this pattern of regulation is also described in the red microalga *C. merolae* (Minoda et al., 2005) but is different from the situation in the green microalga *C. reinhardtii* where post-transcriptional regulation is the rule (Rochaix, 1996).

A striking feature of heterotrophy is the upregulation of many genes encoding proteins of the large (60S) and small (40S) ribosomal subunits (Supplementary Table 4 and Supplementary Figure 2). This suggests that more cytosolic ribosomes are present in heterotrophy than in phototrophy, which would reflect a better fitness of the cells in heterotrophy than in phototrophy in the conditions used here (low CO_2_). This low CO_2_ environment is also reflected by the presence of transcripts of the pseudo-carbon concentration mechanism and the photorespiration in phototrophy. An increase of the expression of genes encoding ribosomal proteins was found when *Galdieria* cells were grown in suboptimal temperature (28°C versus 42°C) and in that case it was considered as a marker of cold stress (Rossoni et al., 2019).

*Galdieria sulphuraria* is well known for the large diversity of its transporters, which are acquired by HGT (Schönknecht et al., 2013). Of the 10 putative acetate transporters of the YaaH family, four are downregulated when cells grown with glucose are compared to cells grown with glycerol and three additional ones are downregulated when DEGs are analyzed between heterotrophy and phototrophy (Supplementary Table 4). A similar situation is found for genes encoding amino acid permeases of the APC and AAAP families and for sugar transporters of the MSF family. Only one gene (Gasu_53180) encoding a putative sugar transporter, is upregulated in glucose (+2.83) compared to glycerol (Supplementary Table 4). Being upregulated in glucose condition compared to glycerol, this suggests that the corresponding transporter could be implicated in glucose assimilation. These gene families of transporters seem thus to be subject to a tight transcriptional regulation depending on the carbon source used for growth. Transcriptional regulation of gene families acquired by HGT, including the transporter families, was also found by Rossoni et al. (2019) in case of cold adaptation. Overall, these results suggest that some of the gene families acquired by HGT are subject to transcriptional regulation in *G. sulphuraria*. Whether this transcriptional regulation is specific to this alga or common amongst the red algae with HGT still needs to be deciphered (Ji et al., 2021).

Considering the biotechnological value of *G. sulphuraria*, the results presented here show that the microalga presents growth parameters similar in glucose and glycerol. This suggests that if the transport sector is based on biodiesel produced by transesterification of triacylglycerols in the near future, glycerol could be used as a source of heterotrophic growth for *G. sulphuraria*. The next step in this field will be to assess to which extent the crude glycerol produced directly by the transesterification process is as efficient as the pure glycerol used in this study for *Galdieria* growth. Knowing that *G. sulphuraria* is able to grow on toxic- and salt-rich media (Reeb and Bhattacharya, 2010), we believe that crude glycerol could also be metabolized efficiently. The presence of pigments when cells are grown with glycerol may represent a problem since they are not desirable for oil production, considering the presence of extra nitrogen that could generate NOx if a mechanical approach is used for oil extraction (Kumar et al., 2015). Our molecular results indicate that there is a strong transcriptional downregulation of genes belonging to photosynthesis, pigment synthesis and also the chloroplast genome in heterotrophy. Still, we cannot conclude about the regulatory mechanism underlying the difference of pigment content between glucose and glycerol-fed cells. It is also worth adding that other targets than oils could also be considered, since *G. sulphuraria* is a good source for nutritional applications due to its high protein content (Graziani et al., 2013).

## Data availability statement

The data presented in this study are deposited in the NCBI repository, accession number PRJNA854810.

## Author contributions

PPS, MC, and AC performed the experiments. All authors designed the experiments, contributed to the analysis and the interpretation of the results, wrote the manuscript, contributed to the article, and approved the submitted version.

## Footnotes

1
http://www.acuf.net/index.php?option=com_content&view=article&id=520:galdieria-sulphuraria-galdieri-merola24&catid=2&Itemid=127&lang=en&Itemid=113

2
http://www.genome.jp/kegg/

3
https://www.ncbi.nlm.nih.gov/sra/PRJNA854810

## Supplementary material

Supplementary Figure 1 Pigments content of *Galdieria sulphuraria* cells grown in heterotrophy in the presence of glucose or glycerol or in phototrophy, during exponential phase (day 2). Bar chart **(A)** shows the pigment content expressed in mg.g DW^–1^. Pigments shown for each condition are, from left to right, zeaxanthin, chlorophyll *a* (Chla), and β-carotene. Bar chart **(B)** shows the phycocyanin content expressed in mg.g^–1^ of soluble proteins. Data are presented as means of three independent biological replicates. Error bars represent standard deviation (±SD).

Supplementary Figure 2 Gene Ontology (GO) and Kyoto Encyclopedia of Genes and Genomes (KEGG) pathway enrichment analysis of DEGs. **(A,B)** GO enrichment analysis of DEGs in heterotrophy in the presence of glucose compared to glycerol **(A)** or in heterotrophy compared to phototrophy **(B)**. Graphs show the 10 most significantly (*p* < 0.05) enriched GO terms at three different levels: biological processes (pink), cellular component (green), and molecular function (blue). Data are expressed in −log10(*p*adj). Labeled values represent the gene count in each pathway. **(C,D)** Significantly enriched KEGG pathways of the DEGs (up and down regulated) in heterotrophy in the presence of glucose compared to glycerol **(C)** or in heterotrophy compared to phototrophy **(D)**. Data are expressed in −log10(*p*adj). Labeled values represent the gene count in each pathway. KEGG, Kyoto Encyclopedia of Genes and Genomes.

Supplementary Table 1 Fatty acid profile and quantification of *Galdieria sulphuraria* cells grown in heterotrophy in the presence of glucose or glycerol. Day 2: exponential phase; Day 4: early stationary phase; Day 8: late stationary phase. SFAs, saturated fatty acids; MUFAs, monounsaturated fatty acids; PUFAs, polyunsaturated fatty acids. Values are expressed in mg g^–1^ DW. The SFA, MUFA, and PUFA classes are expressed in % of the total FAs. The data are presented as the mean ± standard deviation from three independent biological replicates.

Supplementary Table 2 Categorization and abundance of reads. PHOT_1, PHOT_2, and PHOT_3 are samples of *Galdieria sulphuraria* grown in phototrophy without any carbon source. GLU_1, GLU_2, and GLU_3 and samples of *G. sulphuraria* grown in heterotrophy in the presence of 25 mM of glucose. GLY_1, GLY_2, and GLY_3 and samples of *G. sulphuraria* grown in heterotrophy in the presence of 50 mM of glycerol.

Supplementary Table 3 DEG analyses of all the comparisons (GluvsGly, HTvsPHOT, GluvsPHOT, GlyvsPHOT).

Supplementary Table 4 Selected pathways displaying transcript relative variations (glucose versus glycerol in heterotrophy and heterotrophy versus phototrophy). Log2FoldChange values and protein relative abundance only for comparison between heterotrophy and phototrophy. Sheet 1: Photosynthesis, chlorophyll biosynthesis, phycocyanin biosynthesis, carotenoid biosynthesis, Calvin cycle, Carbon concentrating mechanism, Photorespiration; Sheet 2: Mitochondria; Sheet 3: Ribosomes; Sheet 4: Transporters; Sheet 5: Chloroplast genome.

Supplementary Table 5 Kyoto Encyclopedia of Genes and Genomes (KEGG) pathways analyses (GluvsGly, HTvsPHOT, GluvsPHOT, GlyvsPHOT).

Supplementary Table 6 Gene Ontology (GO) analyses (GluvsGly, HTvsPHOT, GluvsPHOT, GlyvsPHOT).
