# Supplementary figures and images for: Cell adaptation of the extremophilic red microalga Galdieria sulphuraria to the availability of carbon sources
Source: Front Plant Sci. 2022 Sep 15;13:978246. doi: 10.3389/fpls.2022.978246 (PMC9520601; doi:10.3389/fpls.2022.978246)

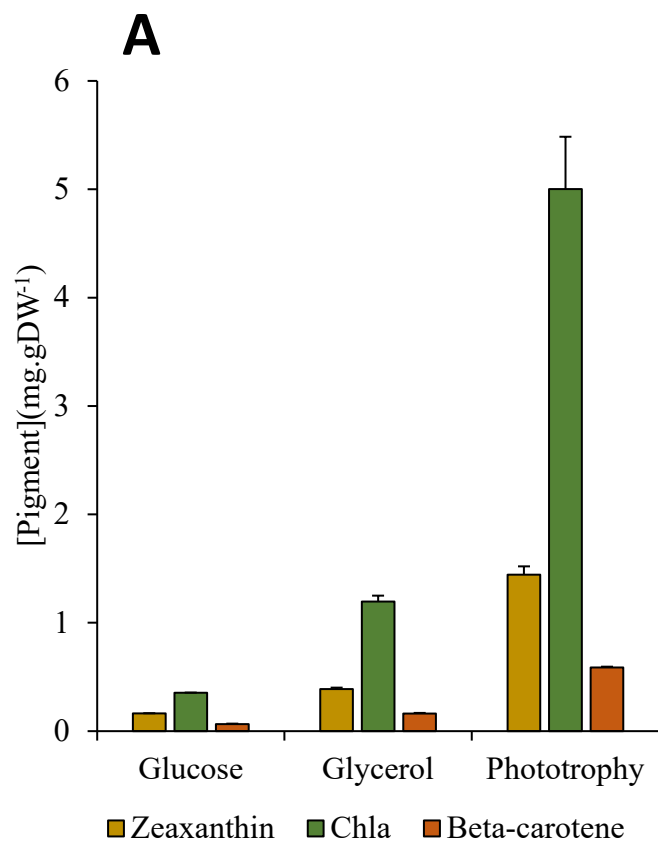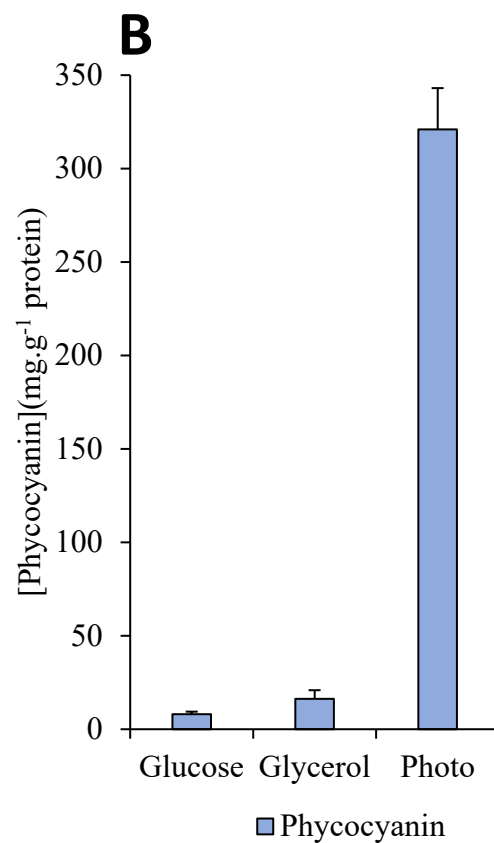

Supplement: Supplementary Figure 1 — Pigments content of Galdieria sulphuraria cells grown in heterotrophy in the presence of glucose or glycerol or in phototrophy, during exponential phase (day 2). Bar chart (A) shows the pigment content expressed in mg.g DW–1. Pigments shown for each condition are, from left to right, zeaxanthin, chlorophyll a (Chla), and β-carotene. Bar chart (B) shows the phycocyanin content expressed in mg.g–1 of soluble proteins. Data are presented as means of three independent biological replicates. Error bars represent standard deviation (±SD). [file Image_1.pdf]
