# Supplementary material for: Cell adaptation of the extremophilic red microalga Galdieria sulphuraria to the availability of carbon sources
Source: Front Plant Sci. 2022 Sep 15;13:978246. doi: 10.3389/fpls.2022.978246 (PMC9520601; doi:10.3389/fpls.2022.978246)
Supplement: Supplementary Figure 2 — Gene Ontology (GO) and Kyoto Encyclopedia of Genes and Genomes (KEGG) pathway enrichment analysis of DEGs. (A,B) GO enrichment analysis of DEGs in heterotrophy in the presence of glucose compared to glycerol (A) or in heterotrophy compared to phototrophy (B). Graphs show the 10 most significantly (p < 0.05) enriched GO terms at three different levels: biological processes (pink), cellular component (green), and molecular function (blue). Data are expressed in −log10(padj). Labeled values represent the gene count in each pathway. (C,D) Significantly enriched KEGG pathways of the DEGs (up and down regulated) in heterotrophy in the presence of glucose compared to glycerol (C) or in heterotrophy compared to phototrophy (D). Data are expressed in −log10(padj). Labeled values represent the gene count in each pathway. KEGG, Kyoto Encyclopedia of Genes and Genomes. [file Image_2.pdf]

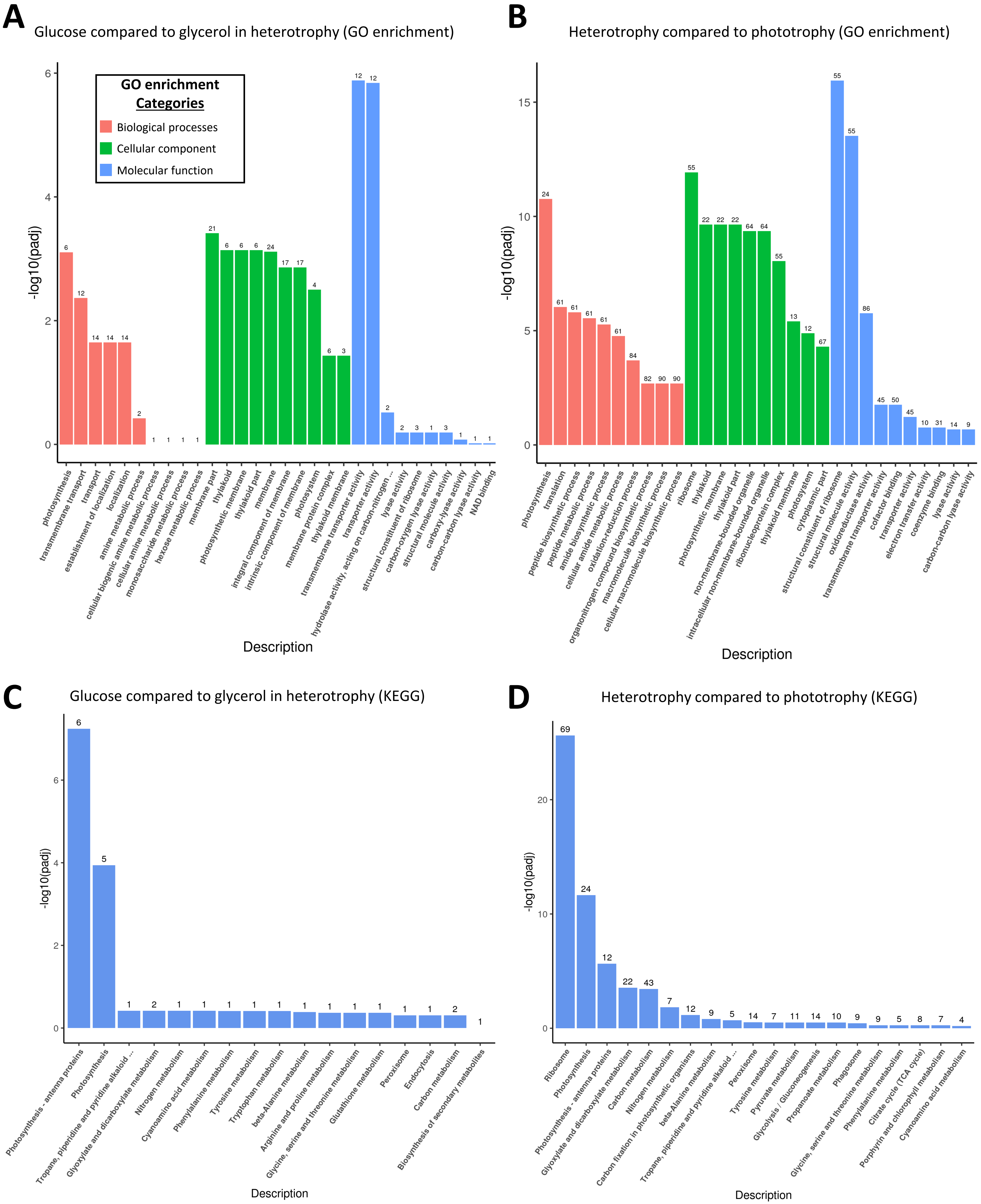
A
Glucose compared to glycerol in heterotrophy (GO enrichment)
GO enrichment Categories
Biological processes
Cellular component
Molecular function
-log10(padj)
Description
photosynthesis
transmembrane transport
transport
establishment of localization
localization
amine metabolic process
cellular biogenic amine metabolic process
cellular amine metabolic process
monosaccharide metabolic process
hexose metabolic process
membrane part
thylakoid
photosynthetic membrane
thylakoid part
membrane
integral component of membrane
intrinsic component of membrane
photosystem
membrane protein complex
thylakoid membrane
transmembrane transporter activity
transporter activity
hydrolase activity, acting on carbon-nitrogen ...
lyase activity
structural constituent of ribosome
carbon-oxygen lyase activity
structural molecule activity
carboxy-lyase activity
carbon-carbon lyase activity
NAD binding
B
Heterotrophy compared to phototrophy (GO enrichment)
-log10(padj)
Description
photosynthesis
translation
peptide biosynthetic process
peptide metabolic process
amide biosynthetic process
cellular amide metabolic process
oxidation-reduction process
organonitrogen compound biosynthetic process
macromolecule biosynthetic process
cellular macromolecule biosynthetic process
ribosome
thylakoid
photosynthetic membrane
thylakoid part
non-membrane-bounded organelle
intracellular non-membrane-bounded organelle
ribonucleoprotein complex
thylakoid membrane
photosystem
cytoplasmic part
structural constituent of ribosome
structural molecule activity
oxidoreductase activity
transmembrane transporter activity
cofactor binding
transporter activity
electron transfer activity
coenzyme binding
lyase activity
carbon-carbon lyase activity
C
Glucose compared to glycerol in heterotrophy (KEGG)
-log10(padj)
Description
Photosynthesis - antenna proteins
Photosynthesis
Tropane, piperidine and pyridine alkaloid ...
Glyoxylate and dicarboxylate metabolism
Nitrogen metabolism
Cyanoamino acid metabolism
Phenylalanine metabolism
Tyrosine metabolism
Tryptophan metabolism
beta-Alanine metabolism
Arginine and proline metabolism
Glycine, serine and threonine metabolism
Glutathione metabolism
Peroxisome
Endocytosis
Carbon metabolism
Biosynthesis of secondary metabolites
D
Heterotrophy compared to phototrophy (KEGG)
-log10(padj)
Description
Ribosome
Photosynthesis
Photosynthesis - antenna proteins
Glyoxylate and dicarboxylate metabolism
Carbon metabolism
Nitrogen metabolism
Carbon fixation in photosynthetic organisms
beta-Alanine metabolism
Tropane, piperidine and pyridine alkaloid ...
Peroxisome
Tyrosine metabolism
Pyruvate metabolism
Glycolysis / Gluconeogenesis
Propanoate metabolism
Phagosome
Glycine, serine and threonine metabolism
Phenylalanine metabolism
Citrate cycle (TCA cycle)
Porphyrin and chlorophyll metabolism
Cyanoamino acid metabolism
