# Supplementary material for: Cell adaptation of the extremophilic red microalga Galdieria sulphuraria to the availability of carbon sources
Source: Front Plant Sci. 2022 Sep 15;13:978246. doi: 10.3389/fpls.2022.978246 (PMC9520601; doi:10.3389/fpls.2022.978246)
Supplement: Supplementary Table 1 — Fatty acid profile and quantification of Galdieria sulphuraria cells grown in heterotrophy in the presence of glucose or glycerol. Day 2: exponential phase; Day 4: early stationary phase; Day 8: late stationary phase. SFAs, saturated fatty acids; MUFAs, monounsaturated fatty acids; PUFAs, polyunsaturated fatty acids. Values are expressed in mg g–1 DW. The SFA, MUFA, and PUFA classes are expressed in % of the total FAs. The data are presented as the mean ± standard deviation from three independent biological replicates. [file Table_1.DOCX]

|  | **Day 2** | | **Day 4** | | **Day 8** | |
| --- | --- | --- | --- | --- | --- | --- |
| **[FA](mg.gDW^-1^)** | **Glucose** | **Glycerol** | **Glucose** | **Glycerol** | **Glucose** | **Glycerol** |
| C14:0 | 0.9 ± 0.2 | 1.3 ± 0.1 | 0.4 ± 0.1 | 1.4 ± 0.2 | 0.7 ± 0.0 | 0.2 ± 0.0 |
| C15:0 | 2.9 ± 0.5 | 3.1 ± 0.1 | 4.0 ± 0.9 | 4.2 ± 0.5 | 3.4 ± 0.1 | 2.2 ± 0.0 |
| C15:1 | / | / | 0.1 ± 0.0 | 0.2 ± 0.0 | 0.6 ± 0.1 | 0.3 ± 0.0 |
| C16:0 | 32.7 ± 7.0 | 35.6 ± 3.3 | 21.7 ± 5.0 | 26.5 ± 2.9 | 21.4 ± 0.4 | 13.5 ± 0.3 |
| C16:1 | / | / | 0.3 ± 0.2 | 0.2 ± 0.1 | 0.3 ± 0.2 | 0.2 ± 0.2 |
| C17:0 | 3.4 ± 1.4 | 2.1 ± 0.2 | 2.6 ± 0.6 | 2.1 ± 0.2 | 2.2 ± 0.0 | 1.9 ± 0.0 |
| C17:1 | / | / | 0.9 ± 0.2 | 0.9 ± 0.1 | 1.2 ± 0.1 | 0.7 ± 0.0 |
| C18:0 | 23.1 ± 4.7 | 22.8 ± 2.3 | 8.9 ± 2.0 | 10.4 ± 0.9 | 5.5 ± 0.1 | 4.3 ± 0.3 |
| C18:1n9t | 1.4 ± 1.3 | 1.8 ± 0.1 | 0.2 ± 0.1 | 0.2 ± 0.1 | 0.4 ± 0.1 | 0.3 ± 0.0 |
| C18:1n9c | 7.9 ± 1.7 | 8.0 ± 0.5 | 8.9 ± 2.0 | 9.7 ± 1.2 | 11.4 ± 0.4 | 6.5 ± 0.3 |
| C18:2n6c | 15.8 ± 4.2 | 17.5 ± 1.4 | 20.4 ± 4.0 | 20.0 ± 2.3 | 20.8 ± 1.0 | 13.2 ± 0.2 |
| C18:3n3 | 23.0 ± 3.7 | 22.9 ± 1.8 | 2.4 ± 0.4 | 1.9 ± 0.3 | 4.5 ± 0.2 | 4.0 ± 0.2 |
| C20:0 | 2.0 ± 0.3 | 1.4 ± 0.7 | 0.2 ± 0.1 | 0.3 ± 0.0 | 0.4 ± 0.1 | 0.3 ± 0.0 |
| C20:1n9 | / | / | 0.3 ± 0.1 | 0.3 ± 0.0 | 0.2 ± 0.0 | 0.1 ± 0.0 |
| C20:2 | / | 6.5 ± 1.3 | 1.3 ± 0.3 | 1.3 ± 0.1 | 1.7 ± 0.0 | 1.4 ± 0.2 |
| Total FA | 113.0 ± 24.0 | 123.1 ± 4.8 | 72.5 ± 15.4 | 79.6 ± 0.6 | 74.6 ± 2.1 | 49.1 ± 1.3 |
| % SFA | 57.4 ± 0.5 | 53.9 ± 3.2 | 52.1 ± 1.5 | 56.5 ± 0.8 | 44.9 ± 0.7 | 45.5 ± 2.7 |
| % MUFA | 8.1 ± 0.7 | 8.0 ± 0.6 | 14.5 ± 0.6 | 14.3 ± 0.3 | 18.9 ± 0.4 | 16.5 ± 0.6 |
| % PUFA | 34.4 ± 0.5 | 38.1 ± 2.7 | 33.4 ± 1.1 | 29.2 ± 0.4 | 36.2 ± 0.5 | 38.0 ± 0.7 |

Fatty acid profile and quantification of *Galdieria sulphuraria* cells grown in heterotrophy in the presence of glucose or glycerol. Day 2: exponential phase; Day 4: early stationary phase; Day 8: late stationary phase (last day of culture). SFAs = saturated fatty acids, MUFAs = monounsaturated fatty acids, PUFAs = polyunsaturated fatty acids. Values are expressed in mg.gDW^−1^. The SFA, MUFA, and PUFA classes are expressed in % of the total FAs. Data are presented as the mean ± standard deviation from three independent biological replicates.
